# Dysregulated circRNA Expression in Juvenile Myoclonic Epilepsy

**DOI:** 10.1007/s12017-026-08928-7

**Published:** 2026-06-06

**Authors:** Fatma Söylemez, Serdal Arslan, Adnan Selim Kimyon, Şükrü Hakan Kaleağası

**Affiliations:** 1 Department of Medical Biology, Faculty of Medicine, Mersin University, Mersin, Turkey; 2 Vocational School of Health Services, Near East University, Nicosia, Cyprus; 3 Department of Neurology, Faculty of Medicine, Mersin University, Mersin, Turkey

**Keywords:** Circular RNA, Epigenetics, Juvenile myoclonic epilepsy, qRT-PCR

## Abstract

Juvenile myoclonic epilepsy (JME) is one of the most common idiopathic generalized epilepsy syndromes, yet its molecular pathogenesis remains incompletely understood. Circular RNAs (circRNAs) are stable non-coding RNAs with important regulatory roles and increasing relevance in neurological disorders. In this study, we investigated for the first time the association between selected circRNAs (hsa_circ_0000218, hsa_circ_0000229, hsa_circ_0000249, hsa_circ_0002010, and hsa_circ_0000143) and JME. Total RNA was isolated from peripheral blood samples of 41 patients with JME and 41 healthy controls, followed by RNase R treatment and quantitative real-time PCR analysis. Expression levels of hsa_circ_0002010 and hsa_circ_0000143 were significantly upregulated in patients with JME compared with controls (both *p* < 0.001), whereas hsa_circ_0000249 was significantly downregulated (*p* < 0.001). No significant differences were observed for hsa_circ_0000218 or hsa_circ_0000229. Sex-specific analyses revealed differential expression patterns, and hsa_circ_0002010 and hsa_circ_0000249 were associated with drug resistance. Our findings suggest that certain circRNAs may be involved in molecular pathways related to JME and may provide preliminary information to inform future studies exploring their potential relevance in JME.

## Introduction

Juvenile myoclonic epilepsy (JME) is a common subtype of idiopathic generalized epilepsy (IGE), accounting for approximately 5–10% of all epilepsy cases (Pietrafusa et al., 2021). It typically manifests during adolescence and is characterized by bilateral myoclonic jerks, predominantly affecting the upper extremities, often occurring shortly after awakening (Jallon & Latour, 2005). The rarity of absence seizures can often lead to epilepsy being excluded from the diagnosis. Seizures usually occur shortly after awakening from sleep, often after sleep deprivation. Despite its relatively well-defined clinical phenotype, delays in diagnosis and inappropriate treatment are common, partly due to overlapping electroencephalographic features and incomplete seizure histories. Approximately 130,000 people die from epilepsy each year and the cost of treating epilepsy patients is rising (Allers et al., 2015; Singh & Sander, 2020). Despite treatment, patients may experience severe complications, including memory loss, suicidal thoughts, and treatment-resistant epilepsy (Laxer et al., 2014). Although antiepileptic drugs are effective in many patients, lifelong treatment is usually required, and a substantial proportion of individuals develop drug resistance. The burden of epilepsy on patients and healthcare systems underscores the need for improved diagnostic and prognostic biomarkers and a deeper understanding of disease mechanisms (Duncan et al., 2022; Proix et al., 2021).

Epigenetic regulation has emerged as a critical contributor to epileptogenesis. Among epigenetic factors, non-coding RNAs—particularly circular RNAs (circRNAs)—have attracted increasing attention due to their stability, evolutionary conservation, and regulatory capacity (Zhai et al., 2024). CircRNAs are found in a wide variety of cell types, tissues, and biobodies and many of them show tissue-specific expression patterns (Salzman et al., 2012; Jeck et al., 2013; Nielsen et al., 2022). CircRNAs can act as microRNA sponges, interact with RNA-binding proteins, modulate transcription and splicing, and, in some cases, encode functional peptides (Cui et al., 2018; Liu & Chen, 2022). Recent studies have implicated circRNAs in various neurological disorders, including epilepsy; however, their role in JME has not yet been explored (Dube et al., 2019; Ghafouri-Fard et al., 2022; Wang et al., 2024; Chen et al., 2023).

Several circRNAs, including hsa_circ_0000218, hsa_circ_0000229, hsa_circ_0000249, hsa_circ_0002010, and hsa_circ_0000143, have been associated with cancer, angiogenesis, and neurodegenerative processes. The potential involvement of these circRNAs in JME remains unknown. Therefore, this study aimed to evaluate the expression patterns of these circRNAs in patients with JME, to explore their possible associations with clinical characteristics, and to generate preliminary data within an epigenetic framework.

## Materials and Methods

### Study Participants

A total of 41 patients diagnosed with JME and 41 age- and sex-matched healthy controls were enrolled. All patients were evaluated at the Neurology Clinic of Mersin University. The control group consisted of individuals without neurological, psychiatric, or systemic diseases. The study was approved by the Clinical Research Ethics Committee of Mersin University (Approval No. 2024/486), and written informed consent was obtained from all participants. All procedures were conducted in accordance with the Declaration of Helsinki.

## RNA Extraction and RNase R Treatment

Peripheral blood samples (2 mL) were collected in EDTA-containing tubes. Total RNA was extracted using the A.B.T. RNA Purification Kit according to the manufacturer’s instructions (A.B.T. Laboratory Industry, Cat: 104-01-05). RNA concentration and quality were assessed using the Promega Quantus™ Fluorometer. To enrich circRNAs, total RNA samples were treated with RNase R, which selectively degrades linear RNAs (MYBIOSOURCE, cat. no. MBS4156446) [11].

## Quantitative Real-Time PCR

Complementary DNA (cDNA) was synthesized using the A.B.T™ cDNA Synthesis Kit with RNase inhibitor (High Capacitiy) (Cat. C03-01-05). Quantitative real-time PCR (qRT-PCR) was performed using A.B.T 2X qPCR SYBR-Green MasterMix (Cat. Q03-01-01) kit. hsa_circ_000284 was used as the endogenous control. Primer sequences are listed in Table 1.

## Statistical Analyses

Sample size calculation was performed using G*Power (version 3.1.9.4) with a power of 85% and a fold-change threshold of 1.2. Statistical analyses were conducted using Student’s t-test or one-way ANOVA, as appropriate. A p value < 0.05 was considered statistically significant. Data analysis was performed using SPSS version 26 and the GeneGlobe Data Analysis Center. Expression levels were calculated using the 2^−ΔΔCt method and the fold change values were calculated using the GeneGlobe Data Analysis Center (Qiagen).

**Table 1:** Primer sequences for qRT-PCR

	Sequence (5’-3’)
hsa_circ_0000218	F: TGGAGTGCAGCTTGAAGGTTTAT R: TTCAGAGCCAAAGCGGGAGA
hsa_circ_0000229	F: GGAAGGGGATATAGAAGACCA R: TTCAGATGGGAGTTTTCGGC
hsa_circ_0000249	F: TCCTACTGTTTGTTGGTGGCA R: TTCAGAAGCATGGTGAGCCA
hsa_circ_0002010	F: AGATGCCAGCTTTGGGTTTG R: GGAATTGGAAAGCAGCGTCA
hsa_circ_0000143	F: GTCTAGAGGGCCAGGACATC R: AAGCCCACAATGAGGAGGAA

RT-qPCR Reverse transcription quantitative polymerase chain reaction, F Forward, R Reverse.

## Results

**Fig. 1:**
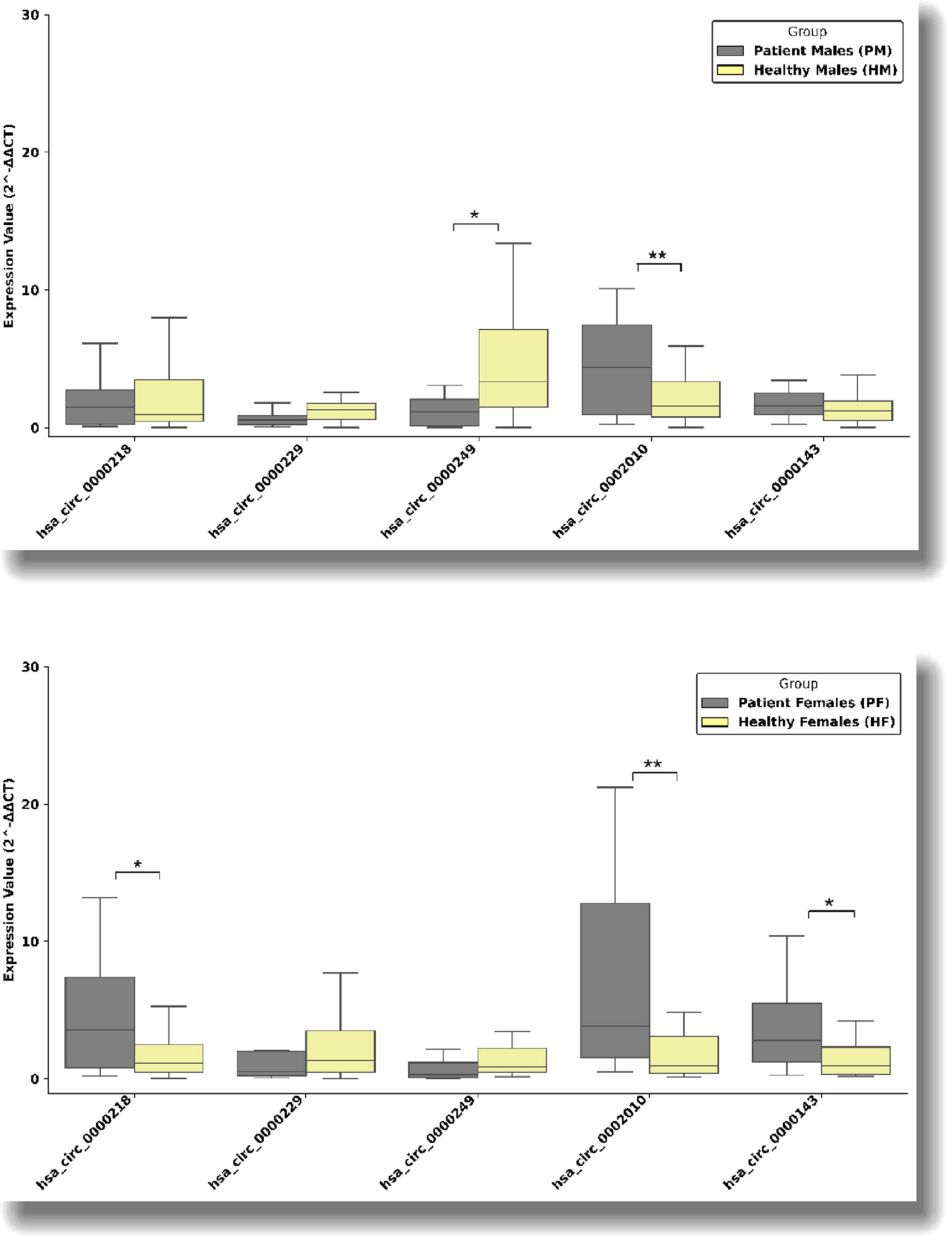
Sex-specific differences in circRNA expression in juvenile myoclonic epilepsy. Relative expression levels of circRNAs were analyzed separately in male and female JME patients compared with sex-matched controls. Results are presented as fold change values. Statistical significance was defined as *p* < 0.05.

**Fig. 2:**
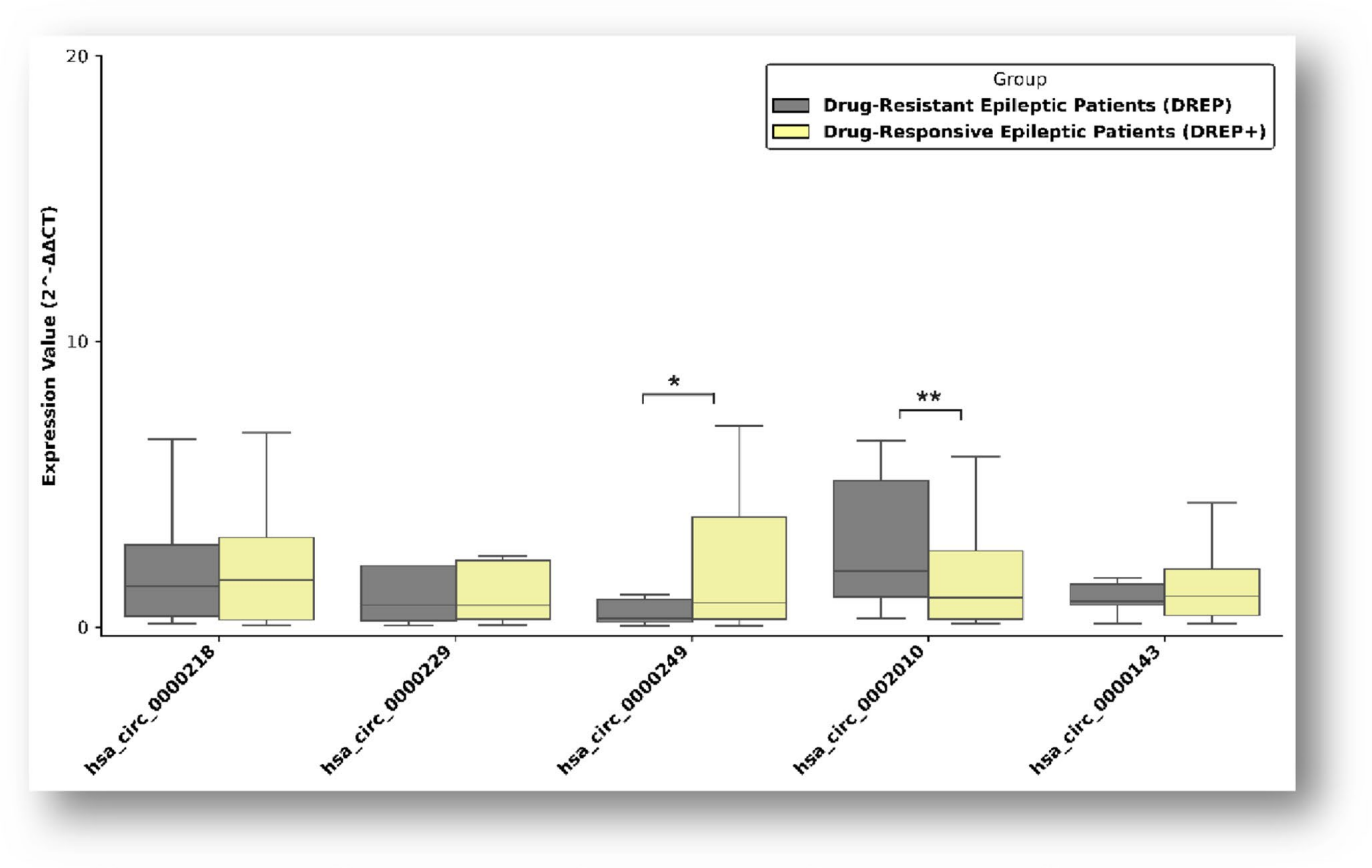
Differential circRNA expression according to drug response status in patients with juvenile myoclonic epilepsy. Comparative analysis of circRNA expression levels between drug-resistant and drug-responsive JME patients. Results are shown as fold change values. Statistical analyses were performed to identify significant differences between groups. *p* < 0.05 indicates statistical significance (*p = 0.04, **p = 0.02)

**Fig. 3:**
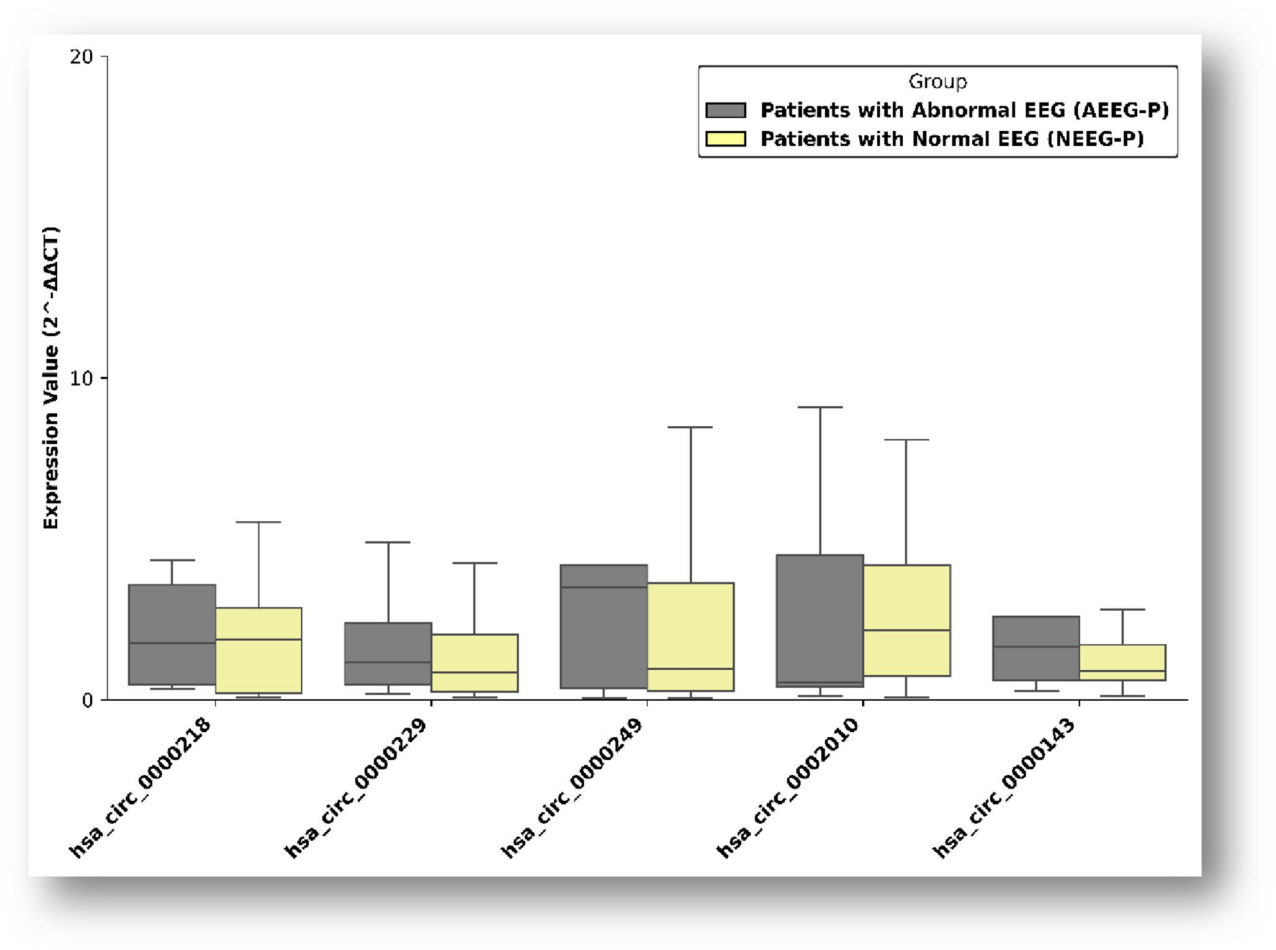
CircRNA expression profiles in juvenile myoclonic epilepsy patients according to electroencephalographic (EEG) findings. Expression levels of selected circRNAs were compared between JME patients with EEG abnormalities and those with normal EEG recordings. Data are presented as fold change. No statistically significant differences were observed unless otherwise indicated (*p* < 0.05)

The mean age of patients with JME was 25 years, and the mean age of controls was 27 years. No significant differences were observed between groups regarding age or sex distribution. Drug resistance was identified in 38.8% of patients. Sex-specific analyses demonstrated distinct expression patterns. In male patients, hsa_circ_0002010 was significantly upregulated (*p* = 0.033), whereas hsa_circ_0000249 was downregulated (*p* < 0.001). In female patients, hsa_circ_0002010, hsa_circ_0000143, and hsa_circ_0000218 showed significant upregulation (*p < 0.001, **p = 0.033) (Fig. 1). Furthermore, hsa_circ_0002010 was significantly upregulated and hsa_circ_0000249 was downregulated in patients with drug-resistant JME, suggesting a potential association with treatment response (*p = 0.04, **p = 0.02) (Fig. 2). CircRNA expression levels did not differ significantly between EEG findings in juvenile myoclonic epilepsy patients (p>0.05) (Fig. 3).

**Fig. 4:**
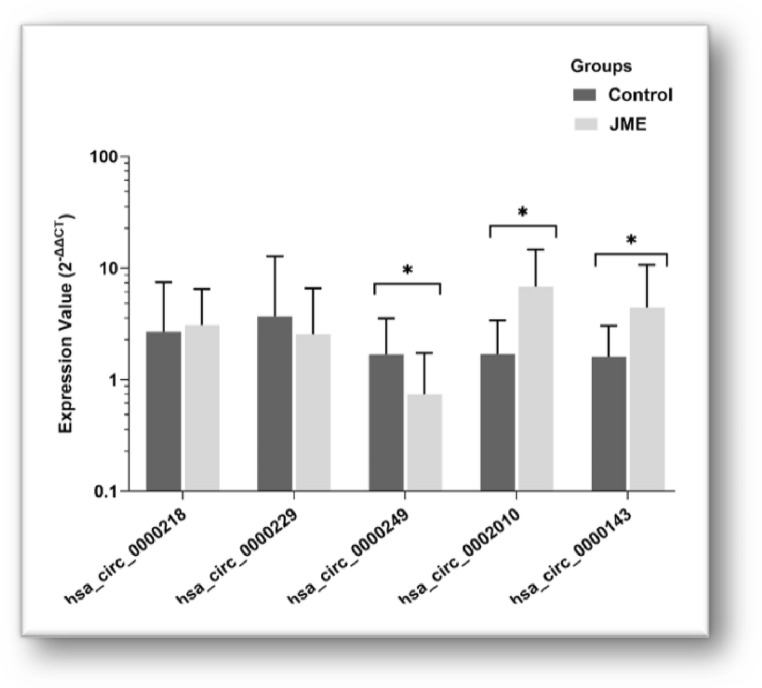
Relative expression levels of circRNAs in juvenile myoclonic epilepsy (JME) patients and healthy controls. Expression levels of hsa_circ_0000218, hsa_circ_0000229, hsa_circ_0000249, hsa_circ_0002010, and hsa_circ_0000143 were measured by quantitative real-time PCR. Data are presented as fold change relative to the control group. Statistical significance was determined using appropriate comparative tests. *p* < 0.05 was considered statistically significant. (*p < 0.001, **p = 0.033)

Expression analysis revealed significant upregulation of hsa_circ_0002010 (3.37-fold, *p* < 0.001) and hsa_circ_0000143 (2.12-fold, *p* < 0.001) in patients with JME compared with controls. In contrast, hsa_circ_0000249 expression was significantly downregulated (3.87-fold, *p* < 0.001). No significant differences were observed for hsa_circ_0000218 or hsa_circ_0000229 (Fig. 4) (Table 2).

**Table 2:** Differential expression of circRNAs in juvenile myoclonic epilepsy according to clinical and demographic variables.

circRNA	JME vs Control (FC)	p value	Drugresistant vs Responsive (FC)	p value		EEG anomaly vs normal EEG (FC)		p value		Female JME vs female control (FC)		p value		Male JME vs male control (FC)		p value
hsa_circ_0000218	1.44	0.663	-1.04	0.840		1.42		0.910		2.49		0.037		-1.09		0.480
hsa_circ_0000229	-1.38	0.477	-1.12	0.570		1.38		0.880		-1.23		0.860		-1.55		0.393
hsa_circ_0000249	-3.87	0.005	-2.48	0.040		1.55		0.380		-3.09		0.090		-6.29		0.002
hsa_circ_0002010	3.37	< 0.001	2.21	0.020		1.13		0.170		4.30		0.007		2.75		0.006
hsa_circ_0000143	2.12	0.006	1.11	0.760		1.61		0.620		2.59		0.033		1.48		0.211

JME, juvenile myoclonic epilepsy; FC, fold change; EEG, electroencephalography. Fold changes represent expression levels relative to the comparison group.

## Discussion

Idiopathic generalized epilepsies account for approximately one-fifth of all epilepsy cases, yet represent less than 1% of epilepsy-related research (Devinsky et al., 2024). Although juvenile myoclonic epilepsy (JME) is the most common generalized epilepsy syndrome, no studies investigating circRNAs in JME have been reported to date. In the present study, we evaluated the expression profiles of hsa_circ_0000218, hsa_circ_0000229, hsa_circ_0000249, hsa_circ_0002010, and hsa_circ_0000143 to address unmet needs in the diagnosis and management of JME.

Our results demonstrated that hsa_circ_0000143 expression was significantly upregulated in patients with JME (*p* < 0.001, fold change [FC] = 2.12). miR-1179, which has multiple predicted binding sites for hsa_circ_0000143, has recently been reported to have biomarker potential in patients with JME (Süsgün et al., 2022). Notably, miR-1179—the only miRNA investigated to date in JME—also contains multiple binding sites for hsa_circ_0002010. In addition, miR-186, which exhibits the highest number of binding sites for hsa_circ_0000143, plays a critical role in the pathogenesis of various cancers by regulating apoptosis and cell survival pathways. Knockdown of circ_0007142 has been reported to suppress malignant biological behaviors in lung adenocarcinoma by modulating the miR-186/FOXK1 axis and attenuating Wnt/β-catenin signaling (Ma et al., 2020). Furthermore, miR-579, which has been linked to alterations in cellular processes including proliferation, migration, apoptosis, and cell cycle regulation in glioblastoma cell lines, contains multiple predicted binding sites for hsa_circ_0000143 (Kalhori et al., 2019). miR-1248 has been identified as a novel prognostic biomarker in supratentorial hemispheric pediatric low-grade gliomas and is associated with disease progression (Catanzaro et al., 2022). This miRNA is involved in key biological processes including cell proliferation, apoptosis, inhibition of DNA repair, and cell growth, and it contains multiple binding sites for both hsa_circ_0000143 and hsa_circ_0002010.

We also observed a significant upregulation of hsa_circ_0002010 expression in the JME group compared with controls (*p* < 0.001, FC = 3.37). miR-1178, which has binding sites for both hsa_circ_0000143 and hsa_circ_0002010, has been shown to be involved in tumor biology. Hsa_circ_0108942 has been reported to be associated with breast cancer progression through regulation of miR-1178-3p/TMED3 axis, and silencing of circ_0108942 has been linked to increased apoptosis and reduced cell proliferation, migration, and invasion in breast cancer cell models (Yang et al., 2023). Conversely, overexpression of miR-1178 has been shown to promote proliferation, migration, and invasion in various cancer types (Cao et al., 2015; Ma et al., 2022). These findings suggest that hsa_circ_0000143 and hsa_circ_0002010 may contribute to the pathogenesis of JME through modulation of miRNA-mediated regulatory networks. Sex-specific analyses revealed that hsa_circ_0002010 expression was upregulated 2.75-fold in male patients with JME (*p* < 0.001). In female patients, hsa_circ_0002010 (*p* < 0.001, FC = 4.30), hsa_circ_0000143 (*p* = 0.033, FC = 2.59), and hsa_circ_0000218 (*p* = 0.033, FC = 2.49) were significantly upregulated. To our knowledge, these findings represent the first report of sex-related expression differences for these circRNAs. Further studies with larger cohorts are required to elucidate the mechanisms underlying these sex-specific expression patterns.

In contrast, hsa_circ_0000249 expression was significantly downregulated in the JME group compared with controls (*p* < 0.001, FC = 3.87) (Fig. 4; Table 1). miR-186, which has the highest number of predicted binding sites for hsa_circ_0000249, has been extensively studied in human cancers and is involved in cell proliferation, apoptosis, cell-cycle regulation, and altered intracellular metabolism (Wang et al., 2019). Studies in glioma have shown that miR-186 is downregulated in the nervous system, consistent with our findings, and contributes to tumor pathogenesis (Wang et al., 2017; Zheng et al., 2015). miR-155, a miRNA known to induce neuronal apoptosis in epilepsy (Duan et al., 2018), also plays a role in regulating inflammatory processes in pediatric epilepsy, particularly in chronic temporal lobe epilepsy (Ashhab et al., 2013). Both miR-155 and tumor necrosis factor-α (TNF-α) levels have been shown to be elevated in children with epilepsy. Additionally, miR-21 has a multifaceted regulatory role in epilepsy progression, promoting neuronal apoptosis or neuronal injury, and exhibits altered expression patterns in children with mesial temporal lobe epilepsy (Lv & Zhou, 2020; Peng et al., 2013). miR-155 and miR-21 contain multiple binding sites for hsa_circ_0000249 and hsa_circ_0002010, suggesting that these circRNAs may influence JME pathogenesis by regulating these miRNAs.

This study provides the first evidence that specific circRNAs are differentially expressed in patients with JME. The observed upregulation of hsa_circ_0002010 and hsa_circ_0000143, together with the downregulation of hsa_circ_0000249, suggests that circRNA-mediated regulatory networks may contribute to the molecular mechanisms underlying JME. These circRNAs are known to interact with microRNAs involved in neuronal survival, apoptosis, inflammation, and synaptic regulation—processes that are central to epileptogenesis (Manna et al., 2022; Hansen et al., 2023). The observed associations with sex and drug resistance further support the clinical relevance of these molecules. The findings of this study should be interpreted in light of certain limitations. The relatively small sample size, primarily due to financial constraints, represents an important limitation. Nevertheless, this study constitutes a preliminary exploratory and descriptive investigation characterizing circRNA expression patterns in JME. Although causal relationships cannot be inferred from the present data, the observed expression patterns may contribute to the existing body of knowledge and may help guide the design of future larger-scale, multicenter studies and functional analyses aimed at elucidating the molecular mechanisms underlying JME pathophysiology.

## Conclusions

In conclusion, this study is the first to indicate the potential relevance of hsa_circ_0002010, hsa_circ_0000143, and hsa_circ_0000249 in juvenile myoclonic epilepsy, and we additionally observed altered expression patterns of hsa_circ_0002010 and hsa_circ_0000249 in patients with drug-resistant JME. The present study provides preliminary evidence of altered expression profiles of these circRNAs and represents the first exploratory and descriptive investigation characterizing circRNA expression patterns in JME. These findings contribute to the emerging literature on epigenetic mechanisms in epilepsy and may help guide future larger-scale, multicenter studies and functional analyses aimed at clarifying the potential clinical relevance of circRNAs in the diagnosis and management of JME.

## Data Availability

The data that support the findings of this study are available on request from the corresponding author. The data are not publicly available dueto privacy or ethical restrictions.
